# Superior fixation of pegged trabecular metal over screw-fixed pegged porous titanium fiber mesh: A randomized clinical RSA study on cementless tibial components

**DOI:** 10.3109/17453674.2011.566139

**Published:** 2011-04-05

**Authors:** Maiken Stilling, Frank Madsen, Anders Odgaard, Lone Rømer, Niels Trolle Andersen, Ole Rahbek, Kjeld Søballe

**Affiliations:** ^1^Department of Orthopedics; ^2^Department of Radiology, Aarhus University Hospital; ^3^Department of Biostatistics, School of Public Health, Aarhus University, Aarhus, Denmark

## Abstract

**Background and purpose:**

Lasting stability of cementless implants depends on osseointegration into the implant surface, and long-term implant fixation can be predicted using radiostereometric analysis (RSA) with short-term follow-up. We hypothesized that there would be improved fixation of high-porosity trabecular metal (TM) tibial components compared to low-porosity titanium pegged porous fiber-metal (Ti) polyethylene metal backings.

**Methods:**

In a prospective, parallel-group, randomized unblinded clinical trial, we compared cementless tibial components in patients aged 70 years and younger with osteoarthritis. The pre-study sample size calculation was 22 patients per group. 25 TM tibial components were fixed press-fit by 2 hexagonal pegs (TM group) and 25 Ti tibial components were fixed press-fit and by 4 supplemental screws (Ti group). Stereo radiographs for evaluation of absolute component migration (primary effect size) and single-direction absolute component migration (secondary effect size) were obtained within the first postoperative week and at 6 weeks, 6 months, 1 year, and 2 years. American Knee Society score was used for clinical assessment preoperatively, and at 1 and 2 years.

**Results:**

There were no intraoperative complications, and no postoperative infections or revisions. All patients had improved function and regained full extension. All tibial components migrated initially. Most migration of the TM components (n = 24) occurred within the first 3 months after surgery whereas migration of the Ti components (n = 22) appeared to stabilize first after 1 year. The TM components migrated less than the Ti components at 1 year (p = 0.01) and 2 years (p = 0.004).

**Interpretation:**

We conclude that the mechanical fixation of TM tibial components is superior to that of screw-fixed Ti tibial components. We expect long-term implant survival to be better with the TM tibial component.

Tibial component loosening remains one of the major causes of failure of cementless total knee arthroplasty (TKA), and the early degree of knee implant migration detected by radiostereometric analysis (RSA) has been shown to predict the long-term survival of the implant (Ryd et al. 1995). With cementless knee arthroplasty, stability is achieved by biological fixation within the first weeks after surgery and the success relies on both correct component position and immediate macrofixation (Soballe et al. 1992). Porous implant surfaces support tissue ingrowth and are generally effective in supplementing bony integration of cementless implants (Bobyn et al. 1982). On the other hand, fibrous integration of tibial knee components leads to reduced strength of mechanical fixation, which is detectable under physiological loads, and to increased early migration measured with RSA, and it may indicate an increased risk of loosening at a later stage (Bellemans 1999). RSA is therefore particularly useful during the first postoperative years (Valstar et al. 2005).

The pore size and structural geometry of coatings in cementless arthroplasty are important factors for early and safe bone ingrowth. Low-porosity coatings, i.e. fiber-metals and beads, may have inferior osseointegration compared to high-porosity coatings with regular interconnecting pores, i.e. trabecular metal (tantalum), which is a newer prosthetic material (Bobyn et al. 1982, Bobyn 1999).

The prosthetic design also influences implant survival and function. A monobloc tibial design offers advantages compared to a modular design in terms of elimination of back-side wear problems and elimination of metallic debris produced by the polyethylene locking mechanism. A pegged tibial design without screw-holes provides an increased surface area for bony fixation and eliminates points where wear debris can directly enter the bone. On the other hand, a modular and screw-fixed design offers a consistent intraoperative macro-fixation (Sumner et al. 1994) with the option of isolated polyethylene liner revision later on (Ryd et al. 1993).

It has been recommended that the fixation of new products for prosthetic surgery should be evaluated by RSA prior to general use (Valstar et al. 2005), and at the time of initiation of this study no clinical RSA data were available for the trabecular metal implant. The aim of this randomized clinical trial (RCT) was to compare the early clinical and migration results (absolute total migration and absolute single-direction migration) in younger osteoarthritic patients treated with two different cementless tibial implants: a new double-pegged trabecular metal tibial component and a well-documented porous, pegged screw-fixed titanium fiber-mesh tibial component.

## Patients and methods

Between November 2003 and April 2005, 50 patients with primary osteoarthritis of the knee were assessed for eligibility to enter this RCT. The study was approved by the Central Denmark Region Committee on Biomedical Research (journal no. 20030119; issue date July 4, 2003) and it was registered with ClinicalTrials.gov (NCT00138853). The trial was performed in compliance with the Helsinki Declaration. Randomization was done in 5 blocks of 10 patients (5 Ti and 5 TM tibial components) by drawing labels in a box, and the labels were then concealed in 50 sequentially numbered closed envelopes. All eligible patients gave their informed consent to participate and were allocated to one of 2 tibial component designs intraoperatively at Aarhus University Hospital, Denmark (after resection of the tibial plateau to ensure adequate bone quality for cementless arthroplasty). 25 patients received a NexGen modular screw-fixed titanium fiber-mesh tibial component and 25 patients received a NexGen trabecular metal monobloc tibial component. The patients were blinded regarding the treatment and implant type during the entire 2-year follow-up period.

### Calculation of sample size

The study was powered for 22 patients per group at a minimal relevant difference of 0.6 mm total translation (power 90%, alpha 0.05, SD 0.6 mm) (Ryd et al. 1993). With expectation of dropouts, 25 patients per group were included.

### Criteria

The inclusion criteria were primary osteoarthritis of the knee, age 18 to 70 years, giving informed consent, and participation in the study with 1 knee per patient. The exclusion criteria were neuromuscular and vascular leg disease, insufficient bone quality for a cementless tibial component, dependency on NSAIDs postoperatively, previous diagnosis of osteoporosis, previous open-wedge tibial osteotomy or other major knee surgery (e.g. cruciate ligament repair), and younger females with a wish to become pregnant within 2 years (coinciding with the follow-up period). All consecutive eligible patients agreed to participate (Table 1).

**Table 1.** Summary of demographics and clinical data at baseline (n = 50). Values are median (range)

Input variables	Titanium fiber-mesh (n = 25)	Trabecular metal (n = 25)
Weight (kg)	85 (73–112)	83 (63–133)
Age (years)	61 (44–70)	64 (37–69)
Implant size	5 (3–7)	5 (3–7)
Sex (male/female)	7/18	11/14
Operated side (right/left)	12/13	15/10
Pain (category 1 through 7)	6 (5–6)	6 (4–7)
Knee flexion (degrees)	110 (90–140)	110 (90–140)
Extension deficit (degrees)	0 (0–10)	0 (0–10)
Knee score (points; max 100)	42 (11–62)	36 (8–62)
Function score (points; max 100)	70 (40–80)	65 (15–90)
Time of surgery (min)	70 (60–100)	68 (55–90)

### Components

**Figure 1.**
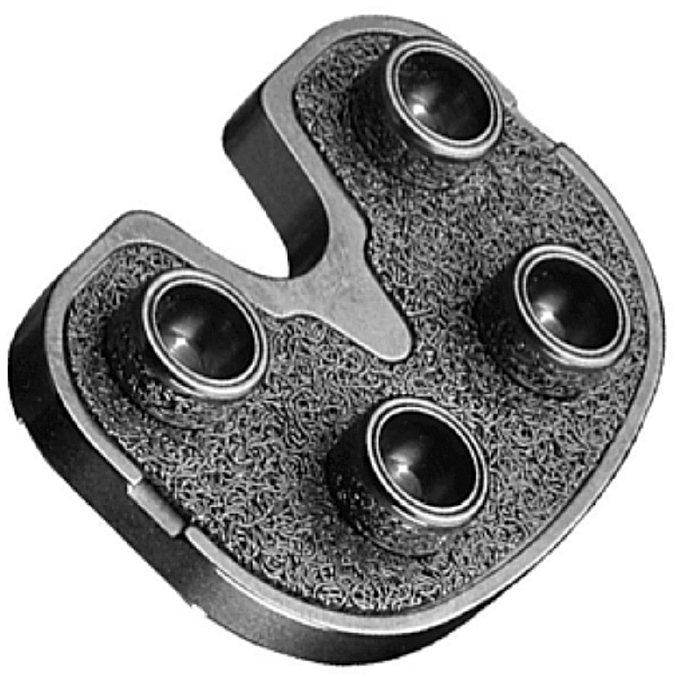
NexGen porous titanium fiber-mesh (Ti) tibial metal-backing with 4 short pegs for screw fixation in the proximal tibia.

**Figure 2.**
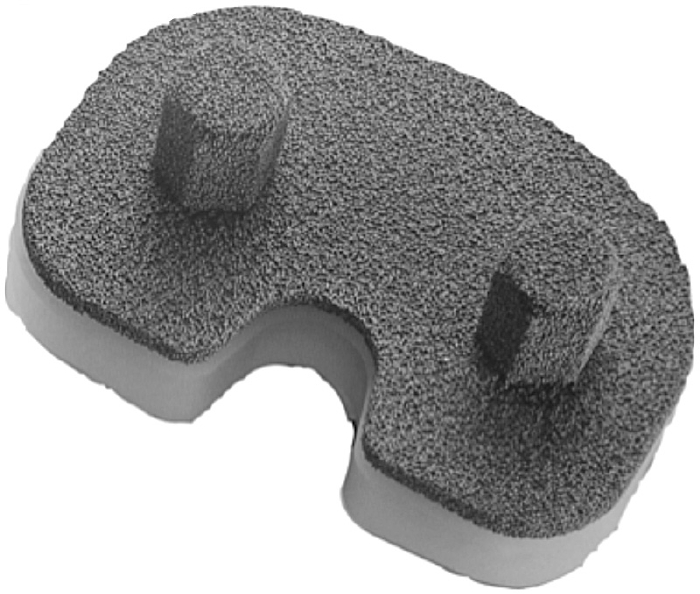
NexGen trabecular metal (tantalum) monobloc with 2 hexagonal pegs for press-fit fixation in the proximal tibia.

**Figure 3.**
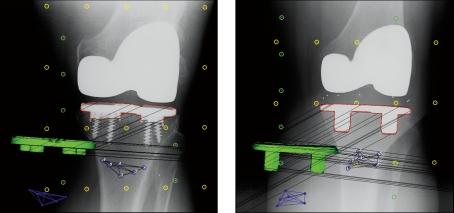
One half of an analyzed model-based RSA image showing the 3-D surface model of the implants (green) with the detected implant contour (red) in the radiographs, and the numbered bone markers (blue) with the corresponding 3-D model (blue). Left: The titanium fiber-mesh screw-fixed tibial component. Right: The double-pegged trabecular metal tibial component.

The NexGen pegged porous titanium fiber-mesh (Ti) metal-backing (Zimmer Inc, Warsaw, IN) was fixed in the tibial bone by 4 cancellous titanium screws through 4 short pegs (Figures 1 and 3). The fiber-metal coatings consist of a titanium alloy with 50% porosity and a pore size of 200–300 μm (Rahbek et al. 2005b). The polyethylene insert is held to the base plate by a peripheral locking mechanism. The NexGen trabecular metal (TM) monobloc (Zimmer) has 2 hexagonal trabecular metal pegs for direct press-fit fixation in the tibial cancellous condyle surface (Figures 2 and 3). Trabecular metal consists of tantalum with 75–80% porosity, a mean pore size of 430 μm, and an elastic modulus similar to that of subcondral bone (Bobyn et al. 1999a, Rahbek et al. 2005a). Both implants are cruciate retaining. The polyethylene was infused directly into the trabecular metal by compression molding to a uniform penetration depth of approximately 1.5 mm. Polyethylene thicknesses of 10 mm and 12 mm and tibial component sizes of 3, 5, and 7 were used. All patellae were resurfaced. The patella and femoral components were NexGen (Zimmer) in all patients, and they were fixed by vacuum-mixed Palacos bone cement with gentamicin (Biomet, Warsaw, IN), making the knee arthroplasty a hybrid.

### Surgery

All patients were operated in a theater with laminar air flow. 4 experienced orthopedic knee surgeons inserted the implants. A tourniquet was applied and an anterior midline incision was used. The posterior cruciate ligament was retained in all cases. In both groups, the proximal tibia was cut using the same extramedullary guide, aiming for a perpendicular cut in the frontal plane and a posterior slope of 3°. Only the guide for the peg-cut differed, having either 2 or 4 holes. The cut surfaces of the patella and femur were cleaned by high-volume/high-pressure irrigation before cementation. The surface of the tibia was protected by a gauze cloth while irrigating the patella and femur. For RSA, we aimed to insert 6 visible 1-mm tantalum beads (Wennbergs Finmek AB, Gunnilse, Sweden) in the proximal tibia. All patients received a draining tube in the joint for approximately 24 h. No navigation equipment was used. All patients were treated prophylactically with a single dose of dicloxacillin (2 g intravenously) and all received prophylactic thrombotic medication (fondaparinux 2.5 g subcutaneously once, for 5–7 days). The patients were mobilized early and were allowed weight bearing as tolerated, but with the assistance of 2 crutches for the first 6 weeks. A CPM machine was used for the first 2 nights. The in-hospital stay varied between 4 and 6 days.

### Follow-up

The patients were seen for clinical examination preoperatively, at 1 year, and 2 years postoperatively. Clinical data collection was conducted unblinded by the 4 surgeons. The validated American Knee Society score (AKSS) (Insall et al. 1989) was used to quantify the functional result (maximum knee score: maximum 100 points; maximum function score: 100 points) and patient satisfaction. Improvement in knee score and in function score has been defined as a change in score of more than 25 points from baseline to follow-up (Danish Knee Arthroplasty Registry, 2006). The patients reported their satisfaction with the result in one of 4 categories according to AKSS: (1) excellent, (2) satisfied, (3) not quite satisfied, or (4) dissatisfied. Before surgery and at 1 and 2 years after surgery, patients rated their knee pain in one of 7 categories according to AKSS: (1) no pain, (2) mild periodic pain, (3) mild pain when climbing stairs, (4) mild pain during normal gait, (5) moderate periodic pain, (6) moderate constant pain, or (7) severe pain.

### Radiostereometric analysis (RSA)

Stereo radiographs were obtained mean 4 (2–7) days postoperatively (reference examination). Subsequent examinations were carried out at 6 weeks, 3 months, 1 year, and 2 years. Stereo radiographic examinations were obtained without weight bearing with the patient supine and the operated knee placed parallel to the calibration box in the same foam positioner at all follow-ups, to position the patient in as identical a position as possible. Thus, the anatomical axis of the leg was parallel with the y-axis of the calibration box. The position and orientation of the global coordinate system in the reference examination defined the direction of implant migration in the follow-up examinations.

A standard RSA setup of 2 synchronized ceiling-fixed roentgen tubes (Arco-Ceil/Medira; Santax Medico, Aarhus, Denmark) with an unfocussed uniplanar carbon calibration box (Box 24; Medis Specials, Leiden, the Netherlands) was used. All stereo radiographs were fully digitized (FCR Profect CS; Fujifilm, Vedbaek, Denmark) (1,760 × 2,140 pixels). The upper limit for mean error rigid body fitting (stable markers used for migration analysis) was 0.5 mm. The mean condition number (dispersion of the bone markers in the tibia) was 56 (SD 23; range 28–117).

Analysis of all stereo radiographs was performed by one observer with Model-Based RSA version 3.1 software (Medis Specials, Leiden, the Netherlands) by laser-scanned reverse-engineered implant models (TNO Industrie en Technick, Leiden, the Netherlands) of 5,000 surface triangles (Kaptein et al. 2003). Implant migration was evaluated using all 5 stereo radiographs with the postoperative stereo radiograph as the reference. The point of measurement was the centre of gravity of the 3-D surface model in relation to the bone markers as the fixed rigid body reference. In some patients, only 3 of 6 markers could be measured due to obstruction by metal or misplaced markers. Interpretation of the signed translations and the rotations is difficult, as values may cancel each other with means/medians close to 0. Since our interest was only in the amount and progression of migration, we only assessed the absolute values statistically. Rotations (implant movement about the axes) were expressed as x-rotation (anterior and posterior tilt), y-rotation (internal and external rotation), and z-rotation (varus and valgus tilt) as well as total rotation (TR = √(x^2^ + y^2^ + z^2^)) (Selvik 1989, Kaptein et al. 2007). Translations (implant movement along the axes) were expressed as x-translation (medial and lateral), y-translation (cranial/lift-off and caudal/subsidence), z-translation (anterior and posterior), and the total translation (TT = √(x^2^ + y^2^ + z^2^)). Total translation is the close equivalent to maximum total point motion (MTPM) (Ryd et al. 1995, Valstar et al. 2005). MTPM was not calculated with the Model-Based RSA software version that we used for analysis. Implants were classified as stable or continuously migrating, as described by Ryd et al. (1995); thus, tibial components with a TT of more than 0.2 mm between 12 and 24 months were considered to be continuously migrating.

**Figure 4.**
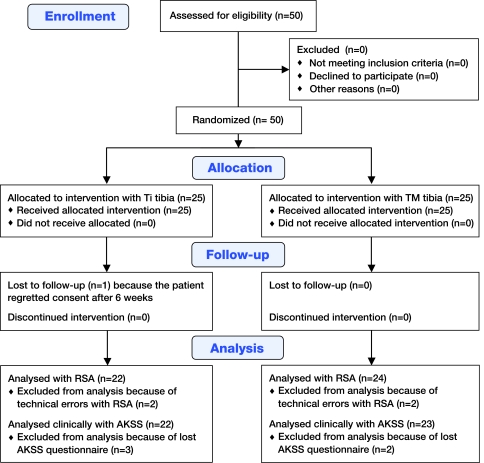
Flow diagram of study.

One patient with a Ti tibia regretted his consent after the 6-week follow-up; he was included for migration analysis until this time point. During follow-up, 4 stereo radiographs could not be analyzed due to technical errors, leaving 47 patients for 6-week analysis (24 Ti and 23 TM), 48 patients for 3-month analysis (23 Ti and 25 TM), 47 patients for 1-year migration analysis (23 Ti and 24 TM), and 46 patients for 2-year migration analysis (22 Ti and 24 TM) (Figure 4).

### Precision of RSA

The repeatability of the measurements was calculated based on double stereo radiographic examinations of 13 patients (7 Ti and 6 TM tibias) at one follow-up (Valstar et al. 2005). The postoperative stereo adiograph was used as the reference in migration analysis of the double examinations, and the expected difference in displacement between the 2 calculations represents the systematic error of the RSA system (bias) and should (optimally) be zero. The standard deviation (SD) represents the precision of the system. The mean signed translations of all axes were all small (< 0.07 mm), and the estimated standard deviations were small (Table 2) and similar to those in other studies (Onsten et al. 1998, Henricson et al. 2008). The mean signed rotations were below 0.04° for the y- and z-axes, but were 0.22° (Ti implant) and 0.07° (TM implant) around the x-axis. The standard deviations for rotation values were 0.27°, 0.25°, and 0.09°, and 0.55°, 0.28°, and 0.10° about the x-, y-, and z-axes for the Ti and the TM tibia, respectively.

**Table 2.** Measurement error of RSA for 13 double-examination stereo radiographs. Signed translations (in mm) for representatives of the 2 tibial components are given. The mean value represents the systematic error, or bias of the system. The standard deviation (SD) represents the precision of the system. The prediction interval (Pi) represents the expected clinical precision

Axis	Titanium fiber-mesh (n = 7)				Trabecular metal (n = 6)			
	x	y	z	Total **^a^**	x	y	z	Total **^a^**
Mean (mm)	–0.02	–0.05	–0.03	0.19	–0.02	0.01	–0.07	0.27
SD (mm)	0.09	0.10	0.26	0.21	0.08	0.08	0.30	0.15
Pi (1.96 × SD)	0.17	0.19	0.51	0.42	0.15	0.16	0.58	0.29
Minimum	0.01	0.01	0.07	0.07	0.03	0.01	0.00	0.03
Maximum	0.33	0.14	0.37	0.52	0.27	0.05	0.11	0.58

**^a^** The total translation was calculated using the 3-D Pythagorean theorem.

The difference in matching of the implant model to the implant contour (model pose estimation) in the radiograph was mean 0.10 mm for the titanium fiber-mesh tibia and 0.17 mm for the TM tibia.

### Statistics

**Figure 5.**
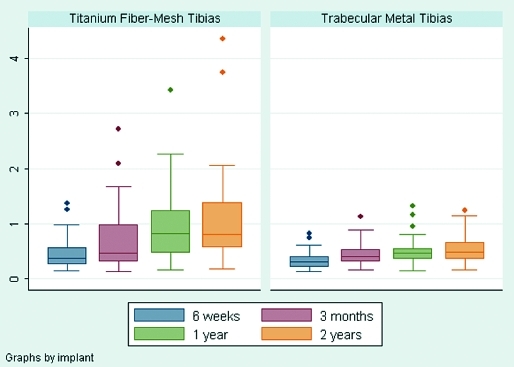
Box plot summarizing the total translation (in mm) of the tibial component in the 2 groups. The line in each box marks the median, the box shows the interquartile range, and the whiskers the tenth and ninetieth percentiles. The TM tibial components migrated less at 1 year (p = 0.01) and 2 years (p < 0.01) than the Ti fiber-metal tibial components. The two patients with Ti tibial components and approximately 4 mm of total translation at 2 years had increasing implant migration at every follow-up after surgery, they had increasing pain score from 1 to 2 years of clinical follow-up, they had decreasing function score and knee score from 1 to 2 years of follow-up, and also the satisfaction level of the patients decreased with the outcome of TKA from 1 to 2 years of follow-up.

All continuous variables were tested for normality (Shapiro-Wilk test (Altman 2009)) and, if normal, were tested further for equal variance (f-test). The groups were then compared by a 2-sample t-test or a 2-sample t-test with unequal variance, as appropriate. If data did not appear to be normally distributed, we tried to achieve normality by transforming the data (log scale and cubic transformation). Data that were not normally distributed were tested by non-parametric tests (Mann-Whitney U-test and Wilcoxon signed-rank test). Categorical data were tested by chi-square test, but cells with observations of 5 and less were tested by Fisher's exact test. All RSA data were assessed statistically as data that were not normally distributed and the medians and interquartile ranges of the TT are presented graphically (Figure 5). For interpretational reasons, however, the absolute translations and rotations are reported as means and standard deviations (Tables 4 and 5). The primary RSA endpoints were the total translation and total rotation values. The secondary RSA endpoints were the individual migrations along and about the single axes. Statistical significance was assumed at p < 0.05. Intercooled Stata version 10.0 (StataCorp, College Station, TX) was used for statistical computations.

## Results

### Clinical results

45 patients of the 50 patients included (22 Ti and 23 TM) were followed clinically for 2 years after TKA. The American Knee Society score (AKSS) form was completed at baseline, and at 1 and 2 years (Tables 1 and 3). No statistically significant differences were found in the clinical data between the 2 groups of patients at 1 and 2 years of follow-up.

**Table 3.** Summary of demographics at the final 2-year follow-up (n = 45)

Input variables	Titanium group (n = 22)	Tantalum group (n = 23)
No pain (category 1) **^a^**	14	17
Mild periodic pain (categories 2 and 3) **^a^**	5	5
Mild pain during gait (category 4) **^a^**	1	0
Moderate pain (categories 5 and 6) **^a^**	2	1
Severe pain (category 7) **^a^**	0	0
Knee flexion (degrees) **^b^**	120 (80–135)	110 (80–135)
Extension deficit (degrees) **^b^**	0	0
Knee score (points; max 100) **^b^**	90 (45–100)	80 (50–100)
Function score (points; max 100) **^b^**	85 (50–100)	90 (60–100)
Patients very satisfied (category 1) **^a^**	15	17
Patients satisfied (category 2) **^a^**	4	3
Patients not quite satisfied (category 3) **^a^**	3	3

**^a^** Patients;

**^b^** Median (range) No statistically significant differences were found between the groups.

All patients rated their functional results as satisfactory (category 1 and 2) in both implant groups at the 1-year follow-up. This became reduced to 19 of 22 patients in the Ti group as compared to 20 of 23 patients in the TM group at 2 years of follow-up, but there were no statistically significant differences between the groups (p = 0.3) (Table 3).

All patients improved in their function scores and knee scores (> 25-point improvements). The mean function scores and knee scores were similar between the groups at the 1-year and 2-year follow-up.

16 patients had an extension deficit at baseline. All regained full extension. Patients with the modular Ti tibial component had a median of 10 degrees more flexion than with the TM tibial component group, but this was not statistically significant.

Before surgery, pain was reported in both groups (categories 4 through 7) (Table 1). All patients registered at 1 year (n = 32) had relief of pain (categories 1, 2, and 3). After 2 years of follow-up (n = 45), 7 patients (5 Ti and 2 TM) reported pain (categories 3–5). 3 of these patients with Ti tibial components had TT migration above 0.4 mm between 12 and 24 months of follow-up and progressive migration throughout the 2 years of follow-up, indicating a poorly fixed tibial component. The one patient with a TM tibial component and pain at 2 years had a stable implant (determined by RSA), but had reduced range of motion due to arthrofibrosis.

### Complications

There were no intraoperative complications and only 2 post-operative complications (1 joint bleeding that was treated non-operatively and 1 arthrofibrosis that was treated by loosening of adherences under anesthesia). At 2.5 years (which was beyond the observation period), 1 Ti tibial component in a patient with a pain rating of 4 and a continuously migrating implant (TT migration of 0.21 mm between 12 months and 24 months of follow-up) was revised, and during surgery the implant was found to be quite clearly loose with no ingrowth of bone into the titanium fiber-mesh.

### RSA results

**Figure 6.**
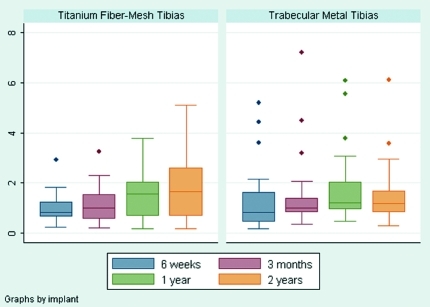
Box plot summarizing the total rotations (in degrees) of the tibial components about the 3 orthogonal axes in the 2 groups. The line in each box marks the median, the box shows the interquartile range, and the whiskers show the tenth and ninetieth percentiles. No significant differences between the groups were found.

At 2 years, the total translation (TT) and total rotation (TR) were 1.14 (SD 1.04, range 0.17–4.4) mm and 1.82 (SD 1.26, 0.17–5.1) degrees for the Ti tibial components, and 0.55 (SD 0.29, 0.16–1.23) mm and 1.54 (SD 1.23, 0.28–6.1) degrees for the TM tibial components. The TT of the Ti tibial components was significantly greater than that of the TM implants at 1 year (p = 0.01) and 2 years (p = 0.004) (Figure 5). Total rotation was similar in the 2 groups (Figure 6). The measured translations and rotations with both implants were well above the limits of precision of the method (Table 2). Most of the migration of the TM tibial components occurred within the first 3 months after surgery, and then, generally speaking, it appeared to stabilize—whereas migration of the screw-fixed titanium fiber-mesh tibial components generally appeared to stabilize after 1 year (Figure 5). However, according to the criteria of Ryd et al. (1995), 12 of 22 patients with Ti tibial components and 2 of 24 patients with TM tibial components had implant migration (TT) of above 0.2 mm between 1 and 2 years of follow-up (p = 0.002) and were classified as having continuous migration.

There was a difference only with x-translation (p = 0.002) when comparing the absolute means of x-, y-, and z-translation and z-rotations separately between the 2 groups at 2 years (Tables 4 and 5).

**Table 4.** Absolute translations (in mm) from the clinical RSA examinations at 2-year follow-up

Axis	Titanium fiber-mesh (n = 22)				Trabecular metal (n = 24)			
	x	y	z	Total **^a^**	x	y	z	Total **^a^**
Mean (mm)	0.54	0.24	0.83	1.14	0.24	0.23	0.35	0.55
SD (mm)	0.42	0.19	1.08	1.04	0.18	0.21	0.29	0.29
Minimum	0.05	0.01	0.00	0.17	0.01	0.01	0.03	0.16
Maximum	1.88	0.62	4.25	4.35	0.73	0.89	1.15	1.23

**^a^** The total translation was calculated using the 3-D Pythagorean theorem.

**Table 5.** Absolute rotations (in degrees) from the clinical RSA examinations at 2-year follow-up

Axis	Titanium fiber-mesh (n = 22)				Trabecular metal (n = 24)			
	x	y	z	Total **^a^**	x	y	z	Total **^a^**
Mean (°)	1.29	0.64	0.71	1.81	1.18	0.43	0.59	1.54
SD (°)	1.24	0.54	0.70	1.26	1.27	0.38	0.41	1.23
Minimum	0.06	0.01	0.02	0.17	0.11	0.05	0.02	0.28
Maximum	4.47	1.79	2.34	5.12	6.08	1.79	1.76	6.10

**^a^** The total translation was calculated using the 3-D Pythagorean theorem.

## Discussion

The role of cementless fixation of knee arthroplasty is still debated and the clinical, histological, and radiological results vary (Hungerford and Kenna 1983, Cook et al. 1988, Effenberger et al. 2001, Lombardi, Jr. et al. 2007, Henricson et al. 2008). The most common reason for revision, apart from polyethylene wear, is aseptic loosening of the tibial component (Robertsson et al. 2001). It is generally accepted that successful bony fixation of cementless implants relies on initial bone-implant contact and stable conditions at the bone-implant interface (Cameron et al. 1973, Soballe et al. 1992). Retrieval studies provide the most accurate description of bone-implant bonding at the time of clinical failure. However, before clinical symptoms and failure, the motion at the bone-implant interface can be assessed as implant migration by radiostereometric analysis, and early and continuous implant migration has been associated with later implant failure (Ryd et al. 1995, Hauptfleisch et al. 2006).

Cementless implants are indicated for younger individuals with good bone quality, because more bone is preserved for later revision surgery. Many variables may have an effect on the course following surgery, and may influence the clinical performance and implant survival. Implant studies in hips and knees have focused on several important details including initial fixation of pegs or screws (Lee et al. 1991, Sumner et al. 1994), bone-implant contact and mechanical strength (Bobyn et al. 1982, Swider et al. 2006), coating porosity and porous sizes (Bobyn et al. 1980, Bobyn 1999, Rahbek et al. 2005a, b), stable and unstable implant conditions (Soballe et al. 1992), and modular or monobloc design (Parks et al. 1998, Rao et al. 2002, Conditt et al. 2004, Macheras et al. 2009).

It has been recommended that new prosthetic designs and materials should be evaluated by RSA before general use (Valstar et al. 2005). The predecessor of the screw-fixed, porous, pegged titanium fiber-metal implant used in our study (the Miller-Galante (MG) total knee prosthesis) was developed in the 1980s and has been thoroughly investigated (Kraay et al. 1991, Ryd et al. 1993, Sumner et al. 1995, Effenberger et al. 2001). Survival rates of 60% at 7 years and of 90% at 5 years, for the MG I and II respectively, have been reported (Effenberger et al. 2001). Bone ingrowth of 40% has been reported for the titanium fiber-mesh on the MG implant (Galante et al. 1971) but as little as 30% bone ingrowth may be sufficient (Stulberg et al. 1991). Ryd et al. (1993) performed the first 2-year follow-up RSA study with the MG tibia and found little postoperative implant migration (mean MTPM of < 1 mm), with the implants usually stabilizing at 1 year. The newer design of tibial component that we investigated was a 2-pegged trabecular metal (tantalum) monobloc. Tantalum is a highly biocompatible, low-modulus transition metal with a high porous appearance similar to cancellous bone (Levine et al. 2006). In the experimental setting as well as in the clinic, trabecular metal has proven successful for early and safe biological fixation (Bobyn et al. 1999a, b, Gruen et al. 2005, Levine et al. 2007).

The major limitation of our study was that it only permitted comparison of implant migration of the 2 tibial components used, but not conclusions regarding contributory factors (i.e. pegs, porosity, screws vs. pegs, and modularity).

Radiostereometric analysis (RSA) of implants using reverse-engineered implant surface models has been described as being a highly accurate method for the evaluation of fixation of tibial components (Kaptein et al. 2003, 2007). The precision in model-based RSA depends on the configuration of the tibial components (the geometry of the outer contour), just as the position of attached implant markers influences the precision of marker-based RSA. Both model-based and marker-based RSA methods may therefore have a different precision for different prosthetic designs (e.g. stemmed vs. flat tibial components), and this should be specified. In our study and for both tibial designs, the implant migrations reported were much higher than the estimated clinical precision based on double-examination RSA. Furthermore, implant migration increased over time, supporting the idea that the migration measured is not the result of methodological imprecision.

The evaluation of implant fixation showed better stability for the pegged trabecular metal tibial components than for the porous pegged titanium fiber-mesh tibial components in assessment of the numeric values of the total translations at 1- and 2-year follow-up. The results support the use of trabecular metal for cementless joint replacement, as do recent studies of migration, mechanical compatibility, and histology with trabecular metal (Levine et al. 2006, Henricson et al. 2008, Dunbar et al. 2009). Screw fixation provides immediate stability in the early period after surgery, with optimal conditions for biological fixation (Sumner et al. 1994), and has proven better than use of 2 pegs without screws (Volz et al. 1988). In theory, the initial rigid fixation of the porous pegged titanium fiber-mesh implant with 4 cancellous screws should limit the risk of early implant motion and development of a fibrous membrane at the bone-implant interface (Lee et al. 1991, Ryd et al. 1993, Sumner et al. 1994). However, in our study screw fixation of the Ti tibial component did not prevent implant migration at 1 and 2 years, which was in contrast to the trabecular metal implant that was fixed press-fit without supplementary screw fixation and stabilized at approximately 3 months.

The migration of trabecular metal tibial components appeared to stabilize after 3 months (Figure 5), which is in accordance with the recent results of Henricson et al. (2008). These authors studied a similar trabecular metal tibial component by RSA but, in contrast to our study, they covered the resected proximal tibia with a thin layer of morselized autograft before insertion of the cementless tibial component. Migration during the first 3 months of observation is a common pattern with well-fixed cementless implants (Nilsson et al. 1991, Onsten et al. 1998, Henricson et al. 2008, Dunbar et al. 2009). Initial migration of cementless implants can be explained as the period of establishment of biological fixation, and at the proximal tibia this mechanism faces challenges including heat necrosis of the bone surface after the cutting process (Toksvig-Larsen et al. 1990) and setting of the tibial component on the slightly irregular, cut tibial surface (Toksvig-Larsen and Ryd 1991). Migration of the titanium fiber-mesh tibial components appeared to stabilize at 1 year on average (Figure 5) but continued at 2 years for several implants. The pore size, the porosity, and the porous-coated area were smaller for the Ti fiber-mesh tibial component than for the TM implant (with no porous coating on the peg tips where the screws penetrated), and a combination of these factors most likely explains the earlier stabilization (faster bone ingrowth) of TM implants (3 months) than with Ti implants (approximately 1 year). A comparison of continuous migration (> 0.2 mm) between the first and the second postoperative year for the 2 types of implants led to high suspicion of fibrous integration and later failure of the Ti tibial components (Ryd et al. 1995, Bellemans 1999). As no implants in either study group were revised during the 2 years of observation, we would need a longer follow-up time to investigate the clinical coherence of larger-scale implant migration and revision with these implants.

The early process of osteoarthritis has been shown to progressively change the microstructure of trabecular bone in the tibia, with increased trabecular thickness and density but reduced mechanical properties of the subchondral cancellous bone (Ding et al. 2003). Although the ageing and osteoarthritic trabeculae align more strongly (parallel) to the tibial longitudinal loading axis (Ding et al. 2002), the mechanical properties decrease with distance from the cartilage and with age (Ding et al. 1998). Thus, it is important that the cutting level on the tibia is of similar thickness when comparing the fixation of 2 different implants. We did not measure the thickness of the cut tibial bone for all patients, but we used the same tibia-cutting guide for both types of implants and we used only 2 thicknesses of polyethylene (10 mm and 12 mm). Thus, we believe that the conditions of bony implant anchorage (tibia level) were similar in the 2 groups.

Trabecular metal has theoretical advantages over titanium fiber-mesh in biological fixation, which may have contributed to the differences in fixation we observed. The high elasticity of trabecular metal may inhibit lift-off at the bone-implant interface and thus protect against the entry of joint fluid containing wear particles, which has been shown to potentiate cell-mediated bone resorption (Rahbek et al. 2000, 2005a). This might also protect against development or persistence of postoperative radiolucent lines at the bone-implant interface, as recently described by Henricson et al. (2008). Radiolucent lines were described as a more common problem in a large series of screw-fixed titanium fiber-mesh (Effenberger et al. 2001). Furthermore, the highly porous cancellous bone-like structure of trabecular metal has been shown to optimize fixation and bone-implant contact in comparison with low porous fiber-metal (Rahbek et al. 2005b).

Our clinical data at 2 years of follow-up showed similar outcome in the 2 groups. However, there was a concurrence in 75% of Ti patients with development of pain at 2 years, no improvement in knee function score, dissatisfaction with the result (category 3), and increased continuous implant migration. One of these patients was revised at 2.5 years, which clearly showed a loose implant with no osseointegration. The mean improvement of 44 points in AKSS in our trabecular metal group was fairly similar to the 51 points that Henricson et al. (2008) recently found. There was a borderline statistically significant median 10-degree difference in flexion at 2 years in favor of the Ti implants; however, these measurements were encumbered with some measurement uncertainty (as they were measured with plastic protractors) that we believe would overshadow the importance of the clinical difference. Although the Ti polyethylene was snap-fit and the TM polyethylene was in-mold, the articulating surfaces of both polyethylenes were identical—as were the femoral components also. Thus, geometric differences between implants do not provide a feasible explanation. Moreover, it is unlikely that the difference in flexion would be related to the RSA results, since increased migration/prosthetic loosening is associated with pain and reduced range of movement.

In conclusion, fixation of the NexGen pegged trabecular metal monobloc tibial component was better than with the NexGen porous, pegged titanium fiber-mesh screw-fixed tibial component as assessed by radiostereometric analysis of the total translation at 1 and 2 years of follow-up. Patients were fully content with the results of both implants at 1 year of follow-up, but after 2 years of follow-up the degree of satisfaction was reduced in both groups. The generalizability of these results can be expected to be high, and we believe that they may be reproduced in any institution with a similar digitized high-quality RSA set-up. Based on previous predictions of reduced implant longevity with early and continuously migrating implants (Grewal et al. 1992, Ryd et al. 1995), we would expect inferior outcome with the pegged titanium fiber-mesh screw-fixed tibial component. Currently, we await the longer-term clinical and radiostereometric results. Although porous tantalum is in its early stages of development, the initial clinical data support the existing experimental data regarding its use as an alternative to traditional orthopedic implant materials.
